# HER2 expression in different cell lines at different inoculation sites assessed by [^52^Mn]Mn-DOTAGA(anhydride)-trastuzumab

**DOI:** 10.3389/pore.2025.1611999

**Published:** 2025-04-29

**Authors:** Toàn Minh Ngô, Adrienn Vágner, Gábor Nagy, Gábor Ország, Tamás Nagy, Zoltán Szoboszlai, Csaba Csikos, Balázs Váradi, György Trencsényi, Gyula Tircsó, Ildikó Garai

**Affiliations:** ^1^ Gyula Petrányi Doctoral School of Clinical Immunology and Allergology, Faculty of Medicine, University of Debrecen, Debrecen, Hungary; ^2^ Department of Nuclear Medicine, Medical Imaging Clinic, Clinical Centre, University of Debrecen, Debrecen, Hungary; ^3^ Scanomed Ltd., Debrecen, Hungary; ^4^ Department of Physical Chemistry, Institute of Chemistry, Faculty of Science and Technology, University of Debrecen, Debrecen, Hungary

**Keywords:** breast cancer, HER2, trastuzumab, positron emission tomography, ^52^Mn

## Abstract

**Purpose:**

Positron emission tomography (PET) hybrid imaging targeting HER2 requires antibodies labelled with longer half-life isotopes. With a suitable radiation profile, ^52^Mn coupled with DOTAGA as a bifunctional chelator is a potential candidate. In this study, we investigated the tumor HER2 specificity and the temporal biodistribution of the [^52^Mn]Mn-DOTAGA(anhydride)-trastuzumab in preclinical models.

**Methods:**

PET/MRI and PET/CT were performed on SCID mice bearing orthotopic and ectopic HER2-positive and ectopic HER2-negative tumors at 4, 24, 48, 72, and 120 h post-injection with [^52^Mn]Mn-DOTAGA(anhydride)-trastuzumab. Melanoma xenografts were included for comparison of specificity.

**Results:**

*In vivo* biodistribution demonstrated strong contrast in HER2-positive tumors, particularly in orthotopic tumors, where uptake was significantly higher than in the blood pool and other organs from 24 h onwards and consistently higher than in ectopic HER2-positive tumors at all time points. Significantly higher tumor-to-blood and tumor-to-muscle ratios were observed in HER2-positive ectopic tumors compared to HER2-negative tumors but only at 4 and 24 h; the differences were likely due to non-specific binding of the tracer. The ratios for orthotopic HER2-positive tumors were significantly higher than those for ectopic HER2-negative tumors and melanoma at all time points. However, the differences between HER2-positive and HER2-negative tumors decreased at later time points.

**Conclusion:**

These results suggest that [^52^Mn]Mn-DOTAGA(anhydride)-trastuzumab demonstrates efficient tumor-to-background contrast, emphasize the higher tumor uptake observed in orthotopic tumors, and highlight the influence of tumor environment characteristics on uptake.

## Introduction

Human Epidermal Growth Factor Receptor 2 (HER2), a transmembrane glycoprotein, plays a pivotal role in cellular signaling through the initiation of cascading events upon either hetero- or homo-dimerization. This activation triggers various proteins, including mitogen-activated protein kinase (MAPK), phosphatidylinositol-4,5-bisphosphate 3-kinase (PI3K), and protein kinase C (PKC). As a result, the HER2 activation regulates many fundamental cellular processes, including proliferation, survival, and migration, thus being a crucial step in tumor growth and invasion [1].

HER2 amplification and overexpression are prevalent across several cancer types, with the highest incidences noted in breast and gastric cancer, representing 20%–30% of cases in these two cancer types [2–4]. Additionally, research studies have reported HER2 involvement in ovarian cancer [5], endometrial cancer [6], esophagus cancer [7], lung cancer [4], and cervical cancer [8]. Moreover, HER2 mutations have also been identified in bladder cancer (more than 10% of cases) and colorectal cancer (5.8% of cases) [9].

Therefore, therapy targeting HER2 has become an essential component of treatment for HER2-positive cancers, particularly in breast and gastric cancer. Trastuzumab, a humanized monoclonal antibody, is the most common HER2-targeted therapy and has significantly improved the overall survival of patients with HER2-enriched breast cancer [10]. Many other strategies, such as targeting different HER2 domains, antibody-drug- conjugates, or tyrosine kinase inhibitors, have recently been approved for treatments; however, these therapies often entail adverse effects, especially cardiovascular risks [11–13].

For this reason, a thorough assessment of HER2 status is required for selecting therapy candidates and monitoring the treatment responses. Currently, fluorescence *in situ* hybridization (FISH) and immunohistochemistry (IHC) are the standard assessment tools, but they are invasive and may not always accurately represent the entire tumor. Consequently, evaluating the intra-tumor and the inter-tumor heterogeneity is suboptimal. Molecular imaging, via positron emission tomography (PET), offers a solution to this challenge by providing a systemic, non-invasive assessment method with both temporal and spatial information.

Trastuzumab-based imaging tracers hold great potential due to their high affinity for HER2 and accurate reflection of the antibody’s biodistribution, paving the way for future radioimmunotherapy dosimetry and personalized treatments. In this context, ^52^Mn, with a prolonged half-life of 5.6 days [14], matches trastuzumab’s *in vivo* half-life (a few days to 23 days, depending on administered dose [15]), enhancing the tracer’s imaging capabilities. Moreover, ^52^Mn can be produced from natural chromium by conventional medical cyclotron using 16–6 MeV bombardment energy [16]. We chose DOTAGA-anhydride as a bifunctional chelator owing to its high reactivity, regioselective anhydride-opening reaction, and the ability to conjugate without needing protection for other chelating ligands or producing unwanted side products [17].

In our study, we utilized [^52^Mn]Mn-DOTAGA-trastuzumab to assess the tumor uptake of HER2-positive and HER2-negative xenografts in preclinical models; melanoma xenografts were included for specificity control. Additionally, we investigated the tumor uptake differences between orthotopic and ectopic HER2-positive tumors.

## Materials and methods

### Chemicals

HPLC-MS grade ACN was from Scharlab Magyarország Ltd. (Debrecen, Hungary). Rotipuran Ultra H_2_O (up H_2_O), Rotipuran Ultra HCl 34% (up HCl), Rotimetic 99.995% anhydrous sodium acetate (NaOAc), and Cellpure HEPES were purchased from Carl Roth GmbH & Co (Germany). Lyophilized Seronorm Human (5 mL) was bought from Sero A/S (Norway). Amicon Ultra (Ultracel – 30K, 0.5 mL) Centrigal Filters were produced by Merck Millipore (Germany). 450 Å, 2.7 µm Bioresolve RP mAb Polyphenyl 2.1 × 50 mm column was used for analytical examinations. Glass macrofiber chromatography paper impregnated with silica gel (iTLC-SG) was supplied by Agilent Technologies (USA).

### ^52^Mn production

The ^52^Mn isotope was produced by proton irradiation with a 14 MeV beam on a natural Cr target via a^52^Cr(p,n)^52^Mn reaction. Based on a previously published method [18], the purification of the radionuclide from CrCl_3_ was performed on AG1-X8 anion-exchange resin using 3% (v/v) HCl in absolute ethanol and 0.1 M HCl solvents. Post-purification was carried out using DGA resin to remove other metal contaminants (e.g., Fe and Cu).

### Synthesis of DOTAGA-trastuzumab

Trastuzumab (Ontruzant^®^, Samsung Bioepis) was purified on a 30 kDa centrifugal filter. Conjugation of pure antibody with DOTAGA-anhydride was carried out at pH 8 in 0.1 M NaHCO_3_ with 20-fold chelator excess. The conjugation efficiency and DOTAGA substitution level were calculated by a UPLC-RA-MS (Waters, USA) system (chelator/antibody ratio: 2.067). DOTAGA-trastuzumab was purified and concentrated to 37 ± 14 mg/mL in up. H_2_O by ultrafiltration.

### Preparation of [^52^Mn]Mn-DOTAGA-trastuzumab

For the preparation of [52Mn]Mn-DOTAGA-trastuzumab, 50 µL of 37 ± 14 mg/mL DOTAGA-trastuzumab solution was added to a mixture of [^52^Mn]MnCl_2_ solution in 0.1 M HCl (29 ± 17 MBq, 100 µL), 0.1 M NaOAc (140 µL), and 0.5 M NaOH (22.5 µL). The reaction mixture was incubated at room temperature for 15 min. Radiochemical purity (RCP) was determined on iTLC-SG by TLC chromatography, using 0.1 M citric acid solution. Each product had an RCP higher than 95%. The reaction mixture was divided into aliquots and diluted to 200 µL portions with 0.9% NaCl solution (molar activity: 12.43 ± 10.85 MBq/mg).

### *In vitro* comparative cell binding study

Our pilot *in vitro* study preliminarily assessed the tracer’s binding differences between HER2+ and HER2- cells lines. Human breast cancer cell lines MDA-MB-HER2+ (a HER2-transduced cell line with high HER2 expression, generated by Dr. György Vereb from the Department of Biophysics and Cell Biology, University of Debrecen, for HER2 research [19, 20]) and MDA-MB-468 (HER2-negative cell line, originally obtained from the American Type Culture Collection (ATCC) Manassas, VA, USA) were gifted to us by the Department of Biophysics and Cell Biology, University of Debrecen. The cells were cultured for 24 h at 36°C in RPMI medium supplemented with 10% fetal bovine serum at a concentration of 0.66 × 10^6^ cells/mL for MDA-MB-HER2+ and 0.52 × 10^6^ cells/mL for MDA-MB-468. The radiopharmaceutical [^52^Mn]Mn-DOTAGA-trastuzumab, with an activity of 1.00 mCi (37 MBq), was added to the cell suspensions, and they were incubated at 36°C for 30, 60, 120, and 180 min. After each incubation period, radioactivity in the cells was measured using a gamma counter. Each cell line was performed in duplicate (n = 2).

### Preclinical model

MDA-MB-HER2+, MDA-MB-468, and murine melanoma cell line B16F10 (American Type Culture Collection) were used for the *in vivo* study. The cells were cultured at 37°C with 5% CO_2_ in DMEM media (from GIBCO Life Technologies, Billings, MT, USA) supplemented with 10% fetal bovine serum and a 1% antibiotic-antimycotic solution (from Merck Life Science Ltd., Budapest, Hungary). The monolayer cell cultures were passaged three times per week.

The study used female mice aged 16–24 weeks, weighing 22.90 ± 4.28 g: CB17 SCID mice used for the breast cancer xenograft study (*n* = 5) and C57BL/6 mice used for the melanoma xenograft study (*n* = 3). All mice (Animalab Ltd., Hungary) were provided with a sterile semi-synthetic diet (VRF1, Akronom Ltd., Hungary) and sterile drinking water. The University of Debrecen’s Ethical Committee for Animal Research in Hungary approved the study protocol (permission number: 16/2020/DEMÁB). Animal handling followed all applicable Hungarian laws and European Union animal welfare regulations taking into account the “4R” principles (based on the principles of reduction, replacement, refinement, and responsibility).

CB17 SCID mice were divided into two groups: the HER2-positive group (*n* = 3) and HER2-negative group (*n* = 2, originally n = 3, one excluded due to death during scanning). The HER2-positive group was injected with 4 × 10^6^ MDA-MB-HER2+ cells mixed thoroughly in 100 μL of saline solution before each injection. This group received two injections: one into the inguinal mammary fat pad and another subcutaneously into the subscapular area. The tumors were then inoculated for 2 weeks after the injection. The HER2-negative group was inoculated subcutaneously under the scapula with 4 × 10^6^ MDA-MB-468 cells suspended in 100 μL of saline solution for the duration of 4 weeks. C57BL/6 mice (*n* = 3) were injected with 2 × 10^6^ B16F10 suspended in 100 μL of saline into the subscapular area and were inoculated for 2 weeks.

### *In vivo* PET imaging

All mice were injected with 3.50 ± 0.59 MBq of [^52^Mn]Mn-DOTAGA-trastuzumab intravenously into the lateral tail veins. Under inhalation anesthesia (induction: 3% isoflurane, maintenance: 2% isoflurane combined with 1–2 L/min O_2_ and 0.8–1 L/min N_2_O) using an anesthesia device (IsoFlo, EICKEMEYER^®^), the HER2-positive groups were scanned with hybrid cameras nanoScan^®^ PET/CT (Mediso Ltd., Hungary), while the HER2-negative and melanoma-bearing mice underwent scans with nanoScan^®^ PET/MRI 1T (Mediso Ltd., Hungary). The scans were conducted using a scanning bed to minimize model movements, while closely monitoring temperature, heart rate, and respiratory rate throughout the procedure. The PET static scans with a duration of 20 min each scan were performed at 4 h, 24 h, 48 h, 72 h, and 120 h post-injection. The anatomical images for localization and attenuation correction maps were obtained by either MRI T1 gradient echo with 0.5 mm slice thickness, 20 ms repetition time, 2.6 ms echo time, and 20° flip angle or CT with 180 projections and 55 kVp X-ray source.

The image reconstruction was done by Nucline software (Mediso Ltd., Hungary) using the Tera-Tomo™ 3D maximum-likelihood expectation-maximization (MLEM) method with attenuation correction, random correction, and scatter correction. Using the InterView™ FUSION software (Mediso Ltd., Hungary), volumes of interest (VOIs) and regions of interest (ROIs) were delineated on the reconstructed images. The VOIs and ROIs were drawn with a 3 mm diameter over the following areas: tumors, mediastinal blood pool, liver lobe, kidney cortex, spleen, lung lobe, pancreas, submandibular salivary gland, knee joint, ovary, lacrimal gland, intestine, urinary bladder, and quadriceps muscle. The measurements were done using the Standardized uptake value (SUV), which was calculated by the software with the formula SUV = [ROI or VOI activity concentration (MBq/mL)]/[injected activity (MBq)/mouse body weight (Gram)]. The measured organ and tumor SUV means to muscle SUV mean ratios were used for investigation. Tumor sizes were assessed using the formula tumor volume (mm^3^) = 0.5 x length (mm) x width (mm) x height (mm) [21].

### Histopathological examination

After the *in vivo* measurements were completed, the mice were humanely euthanized. The tumors were then resected and fixed in 10% neutral buffered formalin. The fixed tumor samples were subsequently sent for histopathological analysis, including staining with Hematoxylin & Eosin and immunohistochemistry, to determine HER2 status.

Tissue sections of 4 μm thickness were cut from representative formaldehyde-fixed paraffin-embedded tissues in one series for all hematoxylin/eosin (H&E) and IHC staining. Routine H&E staining was applied for general histology. For immunohistochemistry, slides were deparaffinized (5 min in xylene), rehydrated, and subjected to a peroxidase-blocking reagent for 5 min (3% hydrogen peroxide was used to block endogenous peroxidase activity). Slides were then washed again and were incubated overnight at 4 °C with Mouse Anti-Human ErbB2/Her2 Monoclonal Antibody (MAB1129; Bio-Techne, Minneapolis, MN, USA) at a dilution of 15 μg/mL. After washing with Tris-buffered saline solution (containing Tween 20, pH 7.6), the primary antibody binding was detected by the anti-Mouse HRP-DAB Cell & Tissue Staining Kit (CTS002; Bio-Techne, Minneapolis, MN, USA) for 5 min. After washing the slides in distilled water, the sections were counterstained with hematoxylin, dehydrated through ethanol and xylene, and cover‐slipped using a xylene‐based mounting medium (Micromount; Leica Biosystems, BioMarker Ltd, Gödöllő, Hungary).

### Statistical analysis

The experimental data were expressed as mean ± standard deviation (SD). Two-way analysis of variance (ANOVA) with post-hoc Tukey’s test was used to analyze the data. A *p*-value less than 0.05 was considered statistically significant. Data analysis and presentation were performed using GraphPad Prism 9.4.1 software and Microsoft Excel.

## Results

### *In vitro* comparative cell binding study

**FIGURE 1:**
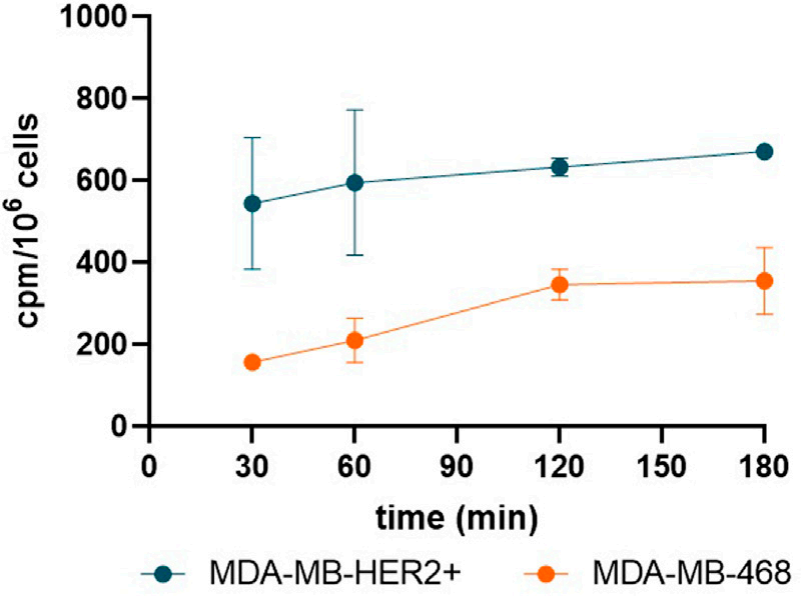
Line graph shows that *in vitro* MDA-MB-HER2+ cells exhibited higher [^52^Mn]Mn-DOTAGA-trastuzumab uptake (counts per minute, cpm) across all time points compared to MDA-MB-468 cells.

The cell binding study demonstrated significant time-dependent uptake of [^52^Mn]Mn-DOTAGA-trastuzumab in both MDA-MB-HER2+ and MDA-MB-468 cell lines. MDA-MB-HER2+ cells consistently exhibited higher counts per minute (cpm) compared to MDA-MB-468 cells, with values increasing from 544.8 ± 161.4 to 671.5 ± 16.4 cpm/10^6^ cells over 180 min, while MDA-MB-468 cells showed a more modest rise, from 156.8 ± 10.8 to 355.4 ± 81.8 cpm/10^6^ cells (Figure 1).

### *In vivo* temporal biodistribution

**FIGURE 2:**
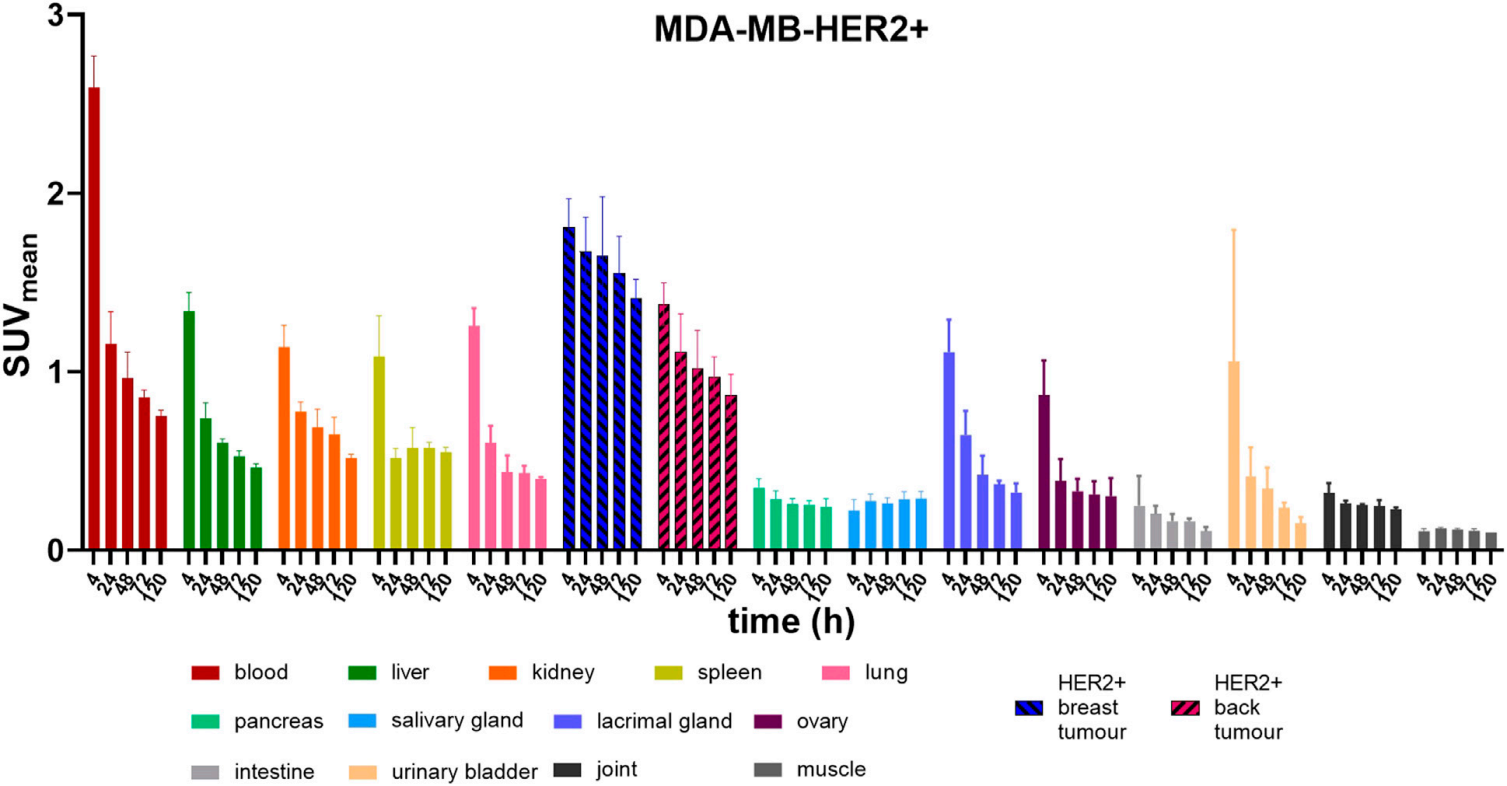
Bar graphs show mean and standard deviation of the 20-min static PET measurements, which were taken at 4, 24, 48, 72, and 120 h post-injection with [^52^Mn]Mn-DOTAGA-trastuzumab into MDA-MB-HER2+ tumour- bearing mice (n = 3) scanned with PET/CT.

*In vivo* biodistribution consistently showed the highest uptake in the blood pool at all time points. Liver, spleen, kidney, and lung all display comparable uptake with similar clearance rate. Low activity was noted in the pancreas, joints, and salivary glands. Initially, elevated activity was observed in the lacrimal glands and ovaries, which gradually cleared in subsequent scans (Figure 2).

In the MDA-MB-HER2+ groups, uptake in HER2-positive tumors was notably higher than in major organs at most time points, particularly evident in breast tumors. Specifically, starting at 24 h post-injection, the activity ratio in breast tumors (SUV_mean_: 1.67 ± 0.19) was significantly higher than that in the blood pool (SUV_mean_: 1.16 ± 0.18) and other major organs (liver, kidney, spleen, and lung) (*p* < 0.01). Conversely, starting from 48 h post-injection, the uptake of ectopic tumors (SUV_mean_: 1.11 ± 0.22) was only significantly higher than that of the liver (SUV_mean_: 0.74 ± 0.09) and lung (SUV_mean_: 0.60 ± 0.10) (*p* < 0.05) and only slightly higher than that of the spleen and kidney in subsequent scans (Figure 2).

### Tumor uptake in HER2-positive orthotopic and ectopic breast xenograft

**FIGURE 3:**
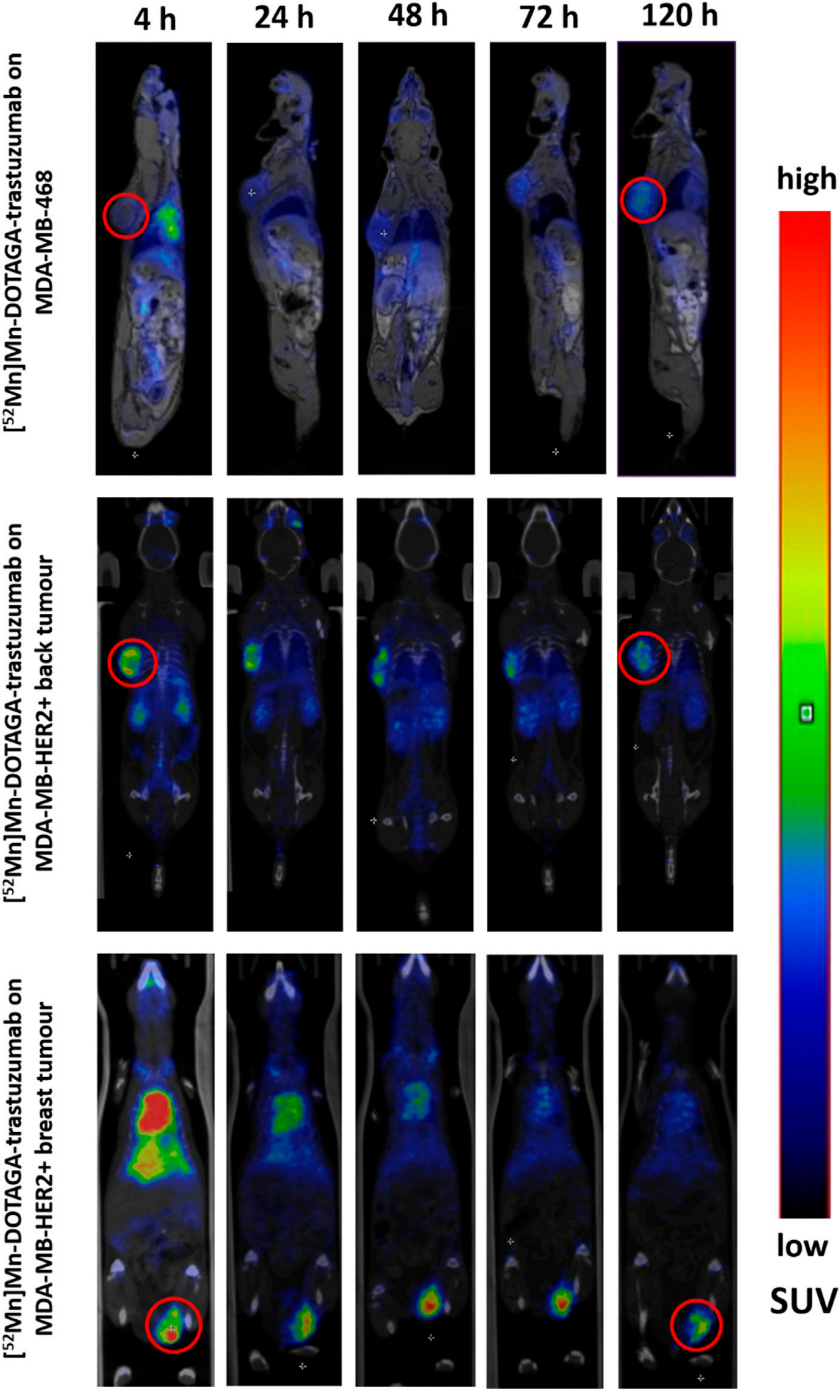
Images of PET/MRI and PET/CT scans were obtained at different time points (4, 24, 48, 72, 120 h) following injection of [^52^Mn]Mn-DOTAGA-trastuzumab into MDA-MB-468 tumour-bearing mice (upper row) and into the MDA-MB-HER2+ tumour-bearing mice (middle and bottom rows displaying the back and breast tumours, respectively). The upper row displays sagittal planes, with the exception of the 48-hour image in coronal plane. The middle and bottom rows display coronal planes. Red circles were used to highlight the MDA-MB-468 back tumour (upper row), the MDA-MB-HER2+ back tumour (middle row), and the MDA-MB-HER2+ breast tumour (bottom row) at 4 h and 120 h time points. There was a prominent high uptake in the HER2-positive orthotopic xenografts, characterised by a particularly good tumor-to-background. Tumor contrast was also visible in the ectopic HER2-positive xenografts but less markedly, with both HER2-positive tumors showing a stronger peripheral uptake compared to the less avid core. In contrast, the HER2-negative xenografts exhibited less pronounced tumor contrast, but this contrast increased at later time points with relatively homogeneous uptake even in the core of the xenografts.

Given the prominent tumor-to-background ratios observed in HER2-positive breast xenografts compared to HER2-positive ectopic tumors based on biodistribution, we investigated the SUV differences between tumor uptake in HER2-positive cell lines inoculated at orthotopic versus ectopic sites. The orthotopic tumors consistently exhibited significantly greater tracer activity than the ectopic tumors at all time points (*p* < 0.01). This difference increased over time and peaked around day 3, with breast tumor SUV_mean_: 1.55 ± 0.21 versus back tumor SUV_mean_: 0.97 ± 0.11 (*p* < 0.0001). Similarly, on imaging, the orthotopic tumors showed significantly higher tumor-to-background contrast, becoming clearly visible from the first time point, whereas the ectopic tumors showed lower contrast, with only peripheral areas being highlighted at most time points (Figure 3).

### Tumor uptake in cell lines with different HER2 expressions

**FIGURE 4:**
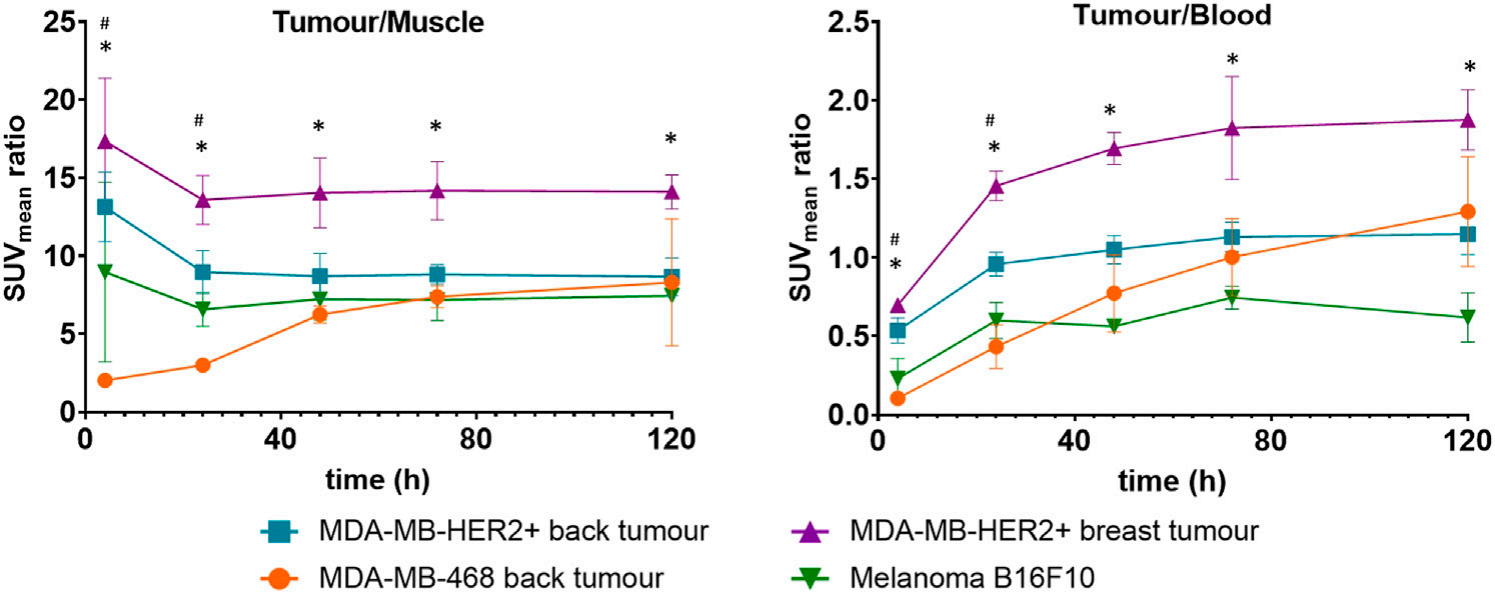
Line graphs represent the mean and standard deviation of the tumor/background ratios of MDA-MB-HER2+ breast (orthotopic) tumors (purple lines), MDA-MB-HER2+ back (ectopic) tumors (blue lines), MDA-MB-468 back tumors (orange lines) in the CB17 SCID mice, and B16F10 tumors (green lines) in the C57BL/6 mice after injection of with [^52^Mn]Mn-DOTAGA-trastuzumab. * indicates significantly higher ratios of breast HER2-positive tumors than all other tumors, # indicates significantly higher ratios of HER2-positive back tumors than HER2-negative tumors.

When comparing the tumor-to-background ratios of the xenografts, the ectopic HER2-positive tumors (tumor-to-blood ratio: 0.54 ± 0.08, tumor-to-muscle ratio: 13.13 ± 2.22) showed significantly higher ratios than the HER2-negative tumors (tumor-to-blood ratio: 0.10 ± 0.02, tumor-to-muscle ratio: 2.02 ± 0.09) at 4 h post-injection (*p* < 0.05); however, these differences began to decrease from 24 h onwards. Significantly higher ectopic HER2-positive tumor-to-blood ratios than ectopic melanoma ratios were observed from 24 h (*p* < 0.05), but the difference was not significant using tumor-to-muscle-ratios (Figure 4).

In contrast, it was evident that the orthotopic HER2-positive tumors consistently exhibited dominance throughout all time points. Specifically, the tumor-to-background ratios of the orthotopic HER2-positive tumors were significantly higher than those of the melanoma at all scan time points and consistently higher than those of the HER2-negative xenografts throughout the study duration, despite an increase in the ratios of the MDA-MB-468 xenografts being observed in the later scans (two-way ANOVA, *p* < 0.05) (Figure 4).

### Histopathological examination

**FIGURE 5:**
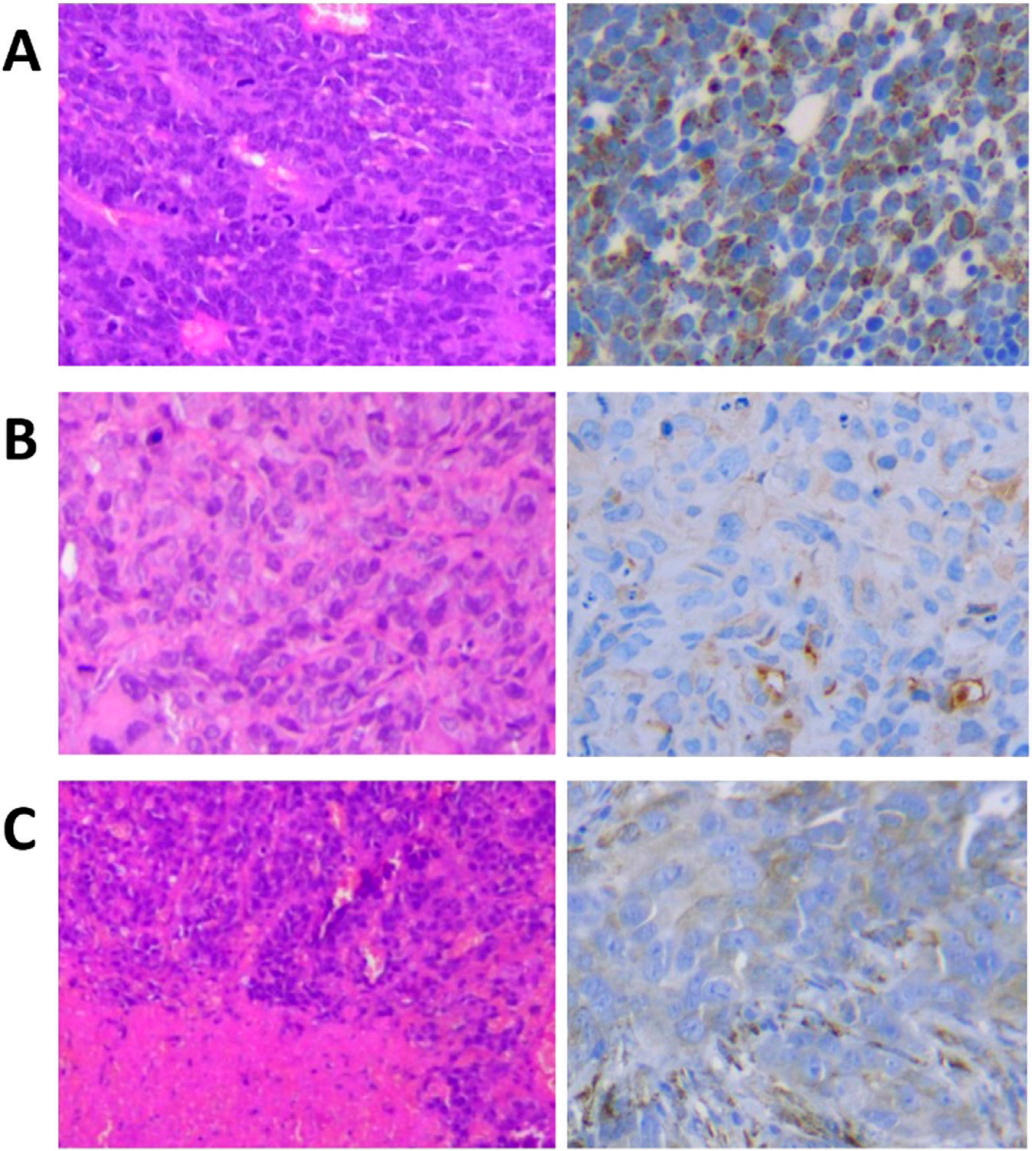
Histopathological H&E staining (left) and the HER2 staining (right) images of the **(A)** MDA-MB-HER2+, the **(B)** MDA-MB-468, and the **(C)** B16F10 after the tumors were resected and fixed with 10% formalin.

Histopathological findings revealed distinct characteristics between the samples. The MDA-MB-HER2+ and B16F10 samples exhibited pronounced necrotic features, particularly in the B16F10 sample. In contrast, the MDA-MB-468 sample demonstrated a complex intertwining of stromal components without necrotic activity (Figure 5).

Our HER2 staining results on the MDA-MB-HER2+ xenografts showed HER2 positivity with varying levels of staining due to necrosis or hypoxia. In contrast, the MDA-MB-468 and B16F10 samples exhibited minimal HER2 staining, perfectly representing HER2-negative tumors (Figure 5).

## Discussion

Given the need for an isotope with a suitable half-life for labelling trastuzumab, many of the radiometals studied still have significant limitations. For instance, ^89^Zr (T_1/2_ = 78.4 h) has been used in [^89^Zr]Zr-trastuzumab studies with promising clinical results [22, 23]. Despite efficient labelling using the chelator DFO, *in vivo* stability of the tracer at later time points is a concern, as free zirconium accumulates in bone, causing unnecessary bone marrow radiation and false-positive bone metastasis. Recent improvements, particularly with DFO* having additional hydroxamic acid, have improved complexation stability *in vivo* [24, 25]. ^64^Cu (T_1/2_ = 12.7 h) has been investigated in [^64^Cu]Cu-trastuzumab studies on patients [26, 27], although its redox activity *in vivo* suggests that further improvements in tracer integrity are needed [24].

^52^Mn, a potential candidate for antibody-based imaging, offers a superior radiation profile compared to ^89^Zr and ^64^Cu. With a T_1/2_ = 5.6 days and a 29% beta-positive emission with low maximum energy (0.575 MeV), ^52^Mn provides a short-tissue penetration and an improved resolution [14]. This makes ^52^Mn an attractive but underexplored option for antibody labelling, with only a few studies investigating its potential, indicating a promising area for further research and development [28–32].

After intravenous injection, the tracer shows an initial high blood uptake due to FcRn-mediated antibody recycling, with moderate liver and spleen uptake driven by FcR-mediated uptake [33] and permeable sinusoidal capillaries [34, 35]. Lung uptake is attributed to the high vascularity [36] and low reflection coefficient of pulmonary tissue, allowing for enhanced tracer uptake [37]. Remarkably, renal activity exceeded typical liver activity for antibody-based tracers, due to the additional carboxylic arm of the tracer (compared to the conventional DOTA chelator), which increases the hydrophilicity of the tracer and consequently elevates the renal excretion [38].

Interestingly, moderate activity was also seen in the ovaries and the lacrimal glands. The physiological expression of HER2 in the ovaries is well-documented [39]. However, since trastuzumab does not cross-react with murine HER2 [40], the ovarian activity may be attributed to non-specific uptake. The lacrimal glands produce protective tears containing immunoglobulins [41], especially under the effect of anesthesia with constant airflow from the breathing mask [42], which causes dry eyes and lens opacity [43]. This was also observed in our scans, resulting in the noticeable tracer uptake in the eyes and lacrimal glands, particularly in SCID mice that lack endogenous immunoglobulins. These findings underscore the advantages of PET imaging in providing a comprehensive whole-body assessment, enabling the detection of unexpected tracer accumulation.

Initial urinary activity in the bladder suggests the rapid excretion of a small portion of unconjugated tracer, as observed in our previous study using unconjugated [^52^Mn]Mn-DOTAGA [28]; nonetheless, low urinary bladder activity in subsequent scans indicates stable conjugation *in vivo*. The kidney, pancreas, salivary glands, and joints are frequently identified as the localized sites of free manganese, as demonstrated in our previous study with [^52^Mn]MnCl_2_ [31] and in other related research [44, 45]. Free manganese is strongly reabsorbed by the kidneys, probably via transporters in proximal tubular cells [46]. In the pancreas, voltage-dependent calcium channels assist in manganese uptake [47] and the metal remains bound to pro-carboxypeptidase B [44]. In our current study, in addition to the stable conjugation, the complexation of the tracer seems to be stable *in vivo,* as suggested by the minimal uptake in the pancreas, salivary glands, and joints without renal reabsorption. Using a similar compound, [^52^Mn]Mn-DOTAGA-p-SCN-trastuzumab, we reported a high stability (RCP maintained above 90% up to day 10) of the ^52^Mn-labelled trastuzumab using the DOTAGA chelator [31].

In our study, both HER2-positive tumors demonstrated high tumor-background contrast; however, significantly higher uptake was observed in the orthotopic tumors compared to the ectopic tumors, possibly due to the microenvironment which favors tracer uptake in orthotopic tumors [48, 49]. This phenomenon corresponds to the better microenvironment characterized by higher microvascular density [50] and increased perfusion [49], which facilitate tracer penetration and promote binding to more specific binding sites.

However, the differences in uptake between MDA-MB-HER2+ and MDA-MB-468 cell lines at the same inoculation site, which diminished over time, are likely due to non-specific binding of the tracer. The larger tumor size in the MDA-MB-468 group (Supplementary Figure S1), leading to increased non-specific uptake [51], is partly responsible for this finding. However, tumor size alone does not explain for the dynamic and clearance of uptake, as aggressive melanoma xenografts, although large, showed lower activity compared to HER2-positive xenografts. Furthermore, melanoma xenografts exhibited a similar uptake pattern to HER2-positive xenografts, in contrast to the MDA-MB-468 group, where uptake increased steadily. This disparity can be due to the fact that both MDA-MB-HER2+ (Figure 5A) and the B16F10 melanoma (Figure 5C) xenografts exhibit necrotic features [52], resulting in decreased overall xenograft uptake [53]. Conversely, the stroma-rich features of MDA-MB-468 xenografts facilitate tracer retention, resulting in slower clearance rate and increased activity over time [54]. Furthermore, the HER2-positive xenograft can show limited tracer uptake due to the binding site barrier as specific binding accumulates in the peripheral parts, reducing uptake in the necrotic/hypoxic core located further from the feeding blood vessels [55], as shown in our *in vivo* images (Figure 3). Whereas MDA-MB-468 tumors with known HER2 phosphorylation at tyrosine Y877 [56] can render the xenografts sensitive to trastuzumab [56, 57].

Our pilot *in vitro* study demonstrates higher tracer uptake in HER2-positive cells; further blocking studies or direct binding assays are required to confirm specific tracer binding. *In vivo,* while the tracer retains some specificity that can differentiate the HER2 positivity in initial scans, tumor uptake includes a high proportion of non-specific binding which is responsible for elevated uptake in HER2-negative tumors at later time points. Astudy using the same compound labelled with radioactive indium, [^111^In]In-DOTAGA(anhydride)-trastuzumab, reported a decrease in the tracer immunoreactivity compared to labelling with the parent DOTA chelator [58]. This reduced immunoreactivity may account for the high non-specific binding observed with our tracer.

Recently, BPPA, a novel bispyclen-based chelator, was used to label trastuzumab with ^52^Mn. The [^52^Mn]Mn-BPPA-trastuzumab showed superior tumor-to-background ratios and earlier detection of HER2 positivity compared to [^52^Mn]Mn-DOTAGA-p-SCN-trastuzumab, though its *in vivo* stability requires improvement [31]. Omweri et al. demonstrated that, with comparable specific activity to [^52^Mn]Mn-BPPA-trastuzumab and enhanced stability, [^52^Mn]Mn-Oxo-DO3A-trastuzumab effectively assessed HER2 status and achieved a higher radiochemical purity yield (RPY) compared to using p-SCN-Bn-DOTA as the chelator [32].

Our study has several limitations. First, there were only two mice in the MDA-MB-468 group, which required the assumption of a normal distribution for the two-way ANOVA analysis. Thus, though our pilot study aimed to generate preliminary insights, further research with larger sample sizes is necessary to provide stronger statistical evidence. Second, discrepancies between PET/CT and PET/MRI can arise from variations in attenuation correction maps [59, 60]. In our study, we also observed systemic differences between these two modalities (Supplementary Tables S1, S2). To address this limitation, we used the tumor-to-background ratio for assessment, aiming to ensure more accurate and reliable results. Using the ratios, such as SUV_mean_ Organ/Muscle, provided reproducible results, as demonstrated by the comparable biodistribution observed in our two breast cancer-bearing groups scanned with different modalities (Supplementary Figure S2). Lastly, we lacked *in vitro* binding assays to further investigate the immunoreactivity or receptor binding characteristics of the tracer.

Based on our findings, we conclude that higher antibody tracer uptake is expected in orthotopic tumors compared to ectopic tumors. However, due to the suboptimal specificity of the tracer, this difference may result from either higher HER2 expression or a more favorable microenvironment at the orthotopic inoculation site, and further investigation is needed to confirm our findings. Several non-specific factors, such as tumor size, necrotic or stromal-rich properties, and the tumor microenvironment, influence imaging outcomes. Despite the need for improved specificity towards HER2, [^52^Mn]Mn-DOTAGA(anhydride)-trastuzumab demonstrates a favorable tumor-to-background ratio and relatively good tracer stability. Additionally, the paramagnetic properties of manganese (II) make it a valuable tool for MRI studies [61], especially in the expanding field of hybrid cameras.

## Conclusion

The study demonstrates high tumor/non-tumor ratios and the relative stability of [^52^Mn]Mn-DOTAGA(anhydride)-trastuzumab and highlights the influence of inoculation sites, tumor characteristics, and the microenvironment on tumor tracer uptake. Despite its ease of production, the tracer exhibits noticeable non-specific binding and requires further improvement in immunoreactivity. Nevertheless, ^52^Mn-labelled trastuzumab has great potential for *in vivo* HER2 assessment as it allows non-invasive longitudinal characterization of HER2 expression in tumors and whole-body imaging to detect unusual tracer accumulation.

## Data Availability

The original contributions presented in the study are included in the article/Supplementary Material, further inquiries can be directed to the corresponding author.
