# Supplementary material for: HER2 expression in different cell lines at different inoculation sites assessed by [52Mn]Mn-DOTAGA(anhydride)-trastuzumab
Source: Pathol Oncol Res. 2025 Apr 29;31:1611999. doi: 10.3389/pore.2025.1611999 (PMC12069034; doi:10.3389/pore.2025.1611999)
Supplement: Supplementary file 1 [file DataSheet1.docx]

**Supporting information**


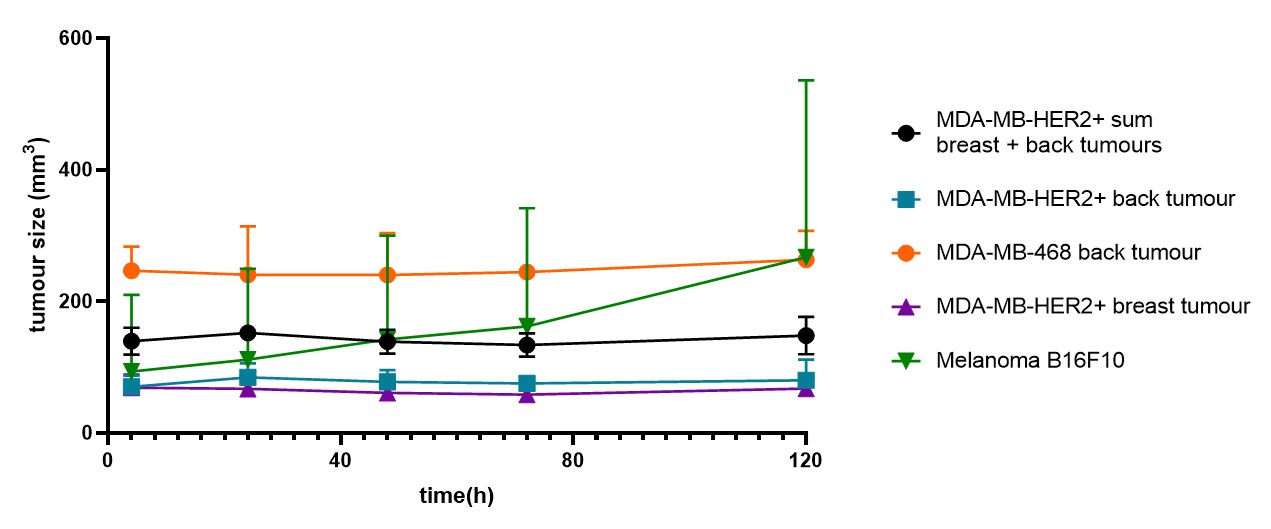


**Figure S1.** The line graph shows progression of tumour size for each tumour group and for the sum of the breast and back HER2+ tumours, data are shown as mean + standard deviation. The larger size of HER2-negative tumours compared to the sum of ectopic and orthotopic HER2-positive tumours can be seen. There was a rapid growth of melanoma tumours that were larger than the sum of HER2-positive tumours on later days.


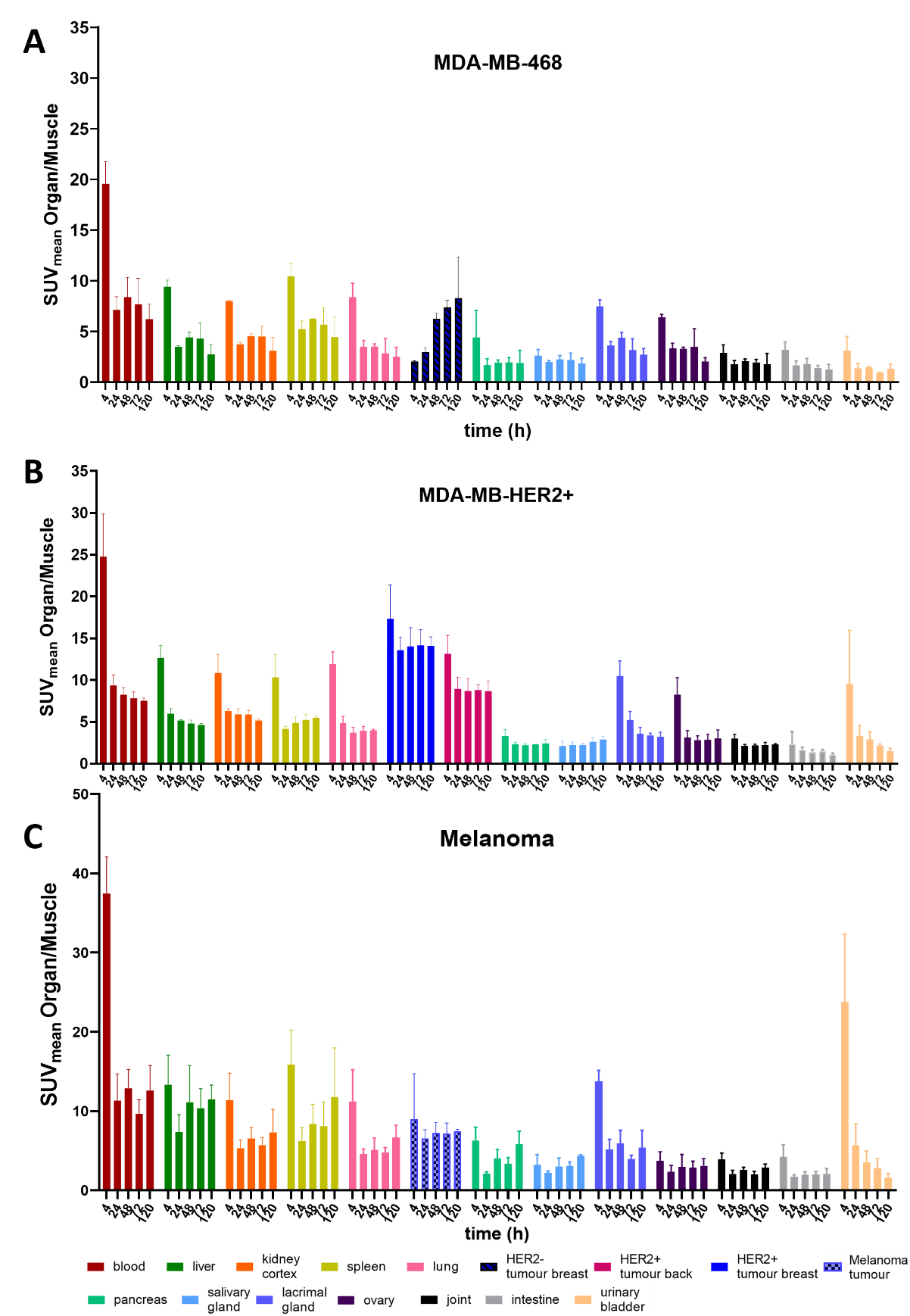


**Figure S2.** Bar graphs show mean and standard deviation of the 20-minute static PET measurements were taken at 4, 24, 48, 72, 120 hours post-injection with [^52^Mn]Mn-DOTAGA-trastuzumab into (**A**) MDA-MB-468 tumour bearing SCID mice (n = 2) scanned with PET/MRI, (**B**) MDA-MB-HER2+ tumour bearing SCID mice (n = 3) scanned with PET/CT, and (**C**) melanoma tumour bearing C57BL/6 mice (n = 3) scanned with PET/MRI. Using SUV_mean_ Organ/Muscle, comparable biodistribution was seen between groups, especially between SCID mice groups, despite scanning with different attenuation maps (PET/CT vs PET/MRI).

**Table S1.** Biodistribuion of [^52^Mn]Mn-DOTAGA-trastuzumab on MDA-MB-HER2+ tumour bearing mice (n=3) using hybrid PET/CT cameras. The data is presented as SUV mean (standard deviation).

|  | 4 h | 24h | 36 h | 72 h | 120 h |
| --- | --- | --- | --- | --- | --- |
| Blood | 2.59 (0.18) | 1.16 (0.18) | 0.97 (0.14) | 0.86 (0.04) | 0.75 (0.03) |
| Liver | 1.34 (0.10) | 0.74 (0.09) | 0.60 (0.02) | 0.53 (0.03) | 0.46 (0.02) |
| Kidney | 1.14 (0.12) | 0.78 (0.06) | 0.69 (0.10) | 0.65 (0.10) | 0.52 (0.02) |
| Spleen | 1.09 (0.23) | 0.52 (0.06) | 0.57 (0.12) | 0.57 (0.03) | 0.55 (0.03) |
| Lung | 1.26 (0.10) | 0.60 (0.10) | 0.44 (0.09) | 0.43 (0.04) | 0.40 (0.01) |
| Pancreas | 0.35 (0.05) | 0.29 (0.05) | 0.26 (0.03) | 0.25 (0.03) | 0.24 (0.05) |
| Salivary gland | 0.22 (0.06) | 0.28 (0.04) | 0.26 (0.03) | 0.29 (0.04) | 0.29 (0.04) |
| Ovary | 0.87 (0.19) | 0.39 (0.12) | 0.33 (0.07) | 0.31 (0.07) | 0.30 (0.10) |
| Lacrimal gland | 1.11 (0.18) | 0.65 (0.14) | 0.42 (0.11) | 0.37 (0.02) | 0.32 (0.05) |
| Joint | 0.32 (0.06) | 0.26 (0.02) | 0.25 (0.01) | 0.25 (0.04) | 0.23 (0.01) |
| Tumour back | 1.38 (0.12) | 1.11 (0.22) | 1.02 (0.21) | 0.97 (0.11) | 0.87 (0.12) |
| Tumour breast | 1.81 (0.16) | 1.67 (0.19) | 1.65 (0.33) | 1.55 (0.21) | 1.41 (0.11) |
| Intestine | 0.25 (0.17) | 0.20 (0.05) | 0.16 (0.04) | 0.16 (0.02) | 0.11 (0.02) |
| Urinary bladder | 1.06 (0.74) | 0.41 (0.16) | 0.34 (0.12) | 0.24 (0.03) | 0.15 (0.03) |
| Muscle | 0.11 (0.02) | 0.12 (0.01) | 0.12 (0.01) | 0.11 (0.01) | 0.10 (0.00) |

**Table S2.** Biodistribuion of [^52^Mn]Mn-DOTAGA-trastuzumab on MDA-MB-468 tumour bearing mice (n=2) using hybrid PET/MRI cameras. The data is presented as SUV mean (standard deviation).

|  | 4 h | 24h | 36 h | 72 h | 120 h |
| --- | --- | --- | --- | --- | --- |
| Blood | 4.37 (0.21) | 2.53 (0.83) | 2.27 (0.53) | 2.08 (0.29) | 2.03 (0.50) |
| Liver | 2.13 (0.48) | 1.22 (0.24) | 1.19 (0.14) | 1.16 (0.18) | 0.88 (0.12) |
| Kidney | 1.80 (0.30) | 1.31 (0.15) | 1.23 (0.06) | 1.23 (0.04) | 0.97 (0.05) |
| Spleen | 2.33 (0.08) | 1.81 (0.01) | 1.69 (0.01) | 1.54 (0.14) | 1.38 (0.04) |
| Lung | 1.91 (0.61) | 1.24 (0.42) | 0.96 (0.06) | 0.76 (0.25) | 0.80 (0.10) |
| Pancreas | 0.95 (0.45) | 0.57 (0.13) | 0.52 (0.07) | 0.54 (0.02) | 0.55 (0.13) |
| Salivary gland | 0.58 (0.04) | 0.70 (0.06) | 0.61 (0.10) | 0.60 (0.06) | 0.60 (0.13) |
| Ovary | 1.44 (0.16) | 1.18 (0.37) | 0.89 (0.04) | 0.93 (0.30) | 0.74 (0.46) |
| Lacrimal gland | 1.68 (0.12) | 1.25 (0.06) | 1.18 (0.15) | 0.85 (0.14) | 0.89 (0.23) |
| Joint | 0.64 (0.08) | 0.61 (0.04) | 0.56 (0.06) | 0.54 (0.03) | 0.52 (0.08) |
| Tumour back | 0.46 (0.09) | 1.04 (0.02) | 1.69 (0.15) | 2.05 (0.22) | 2.53 (0.06) |
| Instestine | 0.71 (0.06) | 0.57 (0.06) | 0.49 (0.15) | 0.39 (0.01) | 0.39 (0.03) |
| Urinary bladder | 0.68 (0.21) | 0.47 (0.09) | 0.39 (0.12) | 0.27 (0.06) | 0.43 (0.06) |
| Muscle | 0.23 (0.04) | 0.35 (0.06) | 0.27 (0.00) | 0.28 (0.06) | 0.35 (0.16) |

**Table S3.** Biodistribution of [^52^Mn]Mn-DOTAGA-trastuzumab on B16F10 tumour bearing C57BL/6 mice (n=3) using hybrid PET/MRI cameras. The data is presented as SUV mean (standard deviation).

|  | 4 h | 24h | 36 h | 72 h | 120 h |
| --- | --- | --- | --- | --- | --- |
| Blood | 6.37 (0.67) | 2.64 (0.65) | 1.93 (0.38) | 1.37 (0.44) | 1.03 (0.39) |
| Liver | 2.21 (0.20) | 1.68 (0.12) | 1.57 (0.16) | 1.41 (0.08) | 0.92 (0.13) |
| Kidney | 1.89 (0.17) | 1.24 (0.16) | 0.98 (0.17) | 0.79 (0.09) | 0.57 (0.18) |
| Spleen | 2.64 (0.14) | 1.43 (0.23) | 1.23 (0.28) | 1.09 (0.23) | 0.90 (0.32) |
| Lung | 1.87 (0.41) | 1.08 (0.23) | 0.75 (0.15) | 0.67 (0.15) | 0.53 (0.14) |
| Pancreas | 1.05 (0.14) | 0.51 (0.13) | 0.61 (0.19) | 0.46 (0.07) | 0.46 (0.02) |
| Salivary gland | 0.53 (0.11) | 0.53 (0.13) | 0.44 (0.09) | 0.43 (0.06) | 0.36 (0.09) |
| Ovary | 0.62 (0.06) | 0.54 (0.12) | 0.41 (0.06) | 0.38 (0.02) | 0.24 (0.02) |
| Lacrimal gland | 2.40 (0.69) | 1.21 (0.29) | 0.89 (0.27) | 0.57 (0.22) | 0.43 (0.15) |
| Joint | 0.67 (0.16) | 0.48 (0.05) | 0.39 (0.12) | 0.27 (0.03) | 0.24 (0.10) |
| Tumour back | 1.40 (0.68) | 1.54 (0.26) | 1.09 (0.22) | 1.00 (0.22) | 0.61 (0.16) |
| Instestine | 0.72 (0.24) | 0.41 (0.08) | 0.31 (0.10) | 0.28 (0.03) | 0.17 (0.08) |
| Urinary bladder | 3.91 (0.82) | 1.31 (0.46) | 0.52 (0.14) | 0.38 (0.13) | 0.14 (0.07) |
| Muscle | 0.17 (0.04) | 0.24 (0.09) | 0.16 (0.06) | 0.14 (0.04) | 0.08 (0.02) |
